# The role of routine flexible sigmoidoscopy in patients presenting with fistula-in-ano: an observational study

**DOI:** 10.1186/s13104-020-05066-6

**Published:** 2020-04-16

**Authors:** Ruvindu Hasamal Waidyasekera, Umesh Jayarajah, Dharmabandhu Nandadeva Samarasekera

**Affiliations:** Department of Surgery, Faculty of Medicine, University of Colombo, P.O. Box 271, Kynsey Road, Colombo 8, Western Province Sri Lanka

**Keywords:** Anal fistula, Fistula-in-ano, Flexible sigmoidoscopy, Yield

## Abstract

**Objective:**

Flexible sigmoidoscopy is useful to look for an underlying aetiology in fistula-in-ano. This study was aimed to assess the yield of routine flexible sigmoidoscopy in patients presenting with fistula-in-ano. A retrospective analysis of 159 consecutive patients with fistula-in-ano who underwent routine flexible sigmoidoscopy was performed. Sigmoidoscopy findings were recorded on a standard uniform format using a computer database. Those with a known aetiology were excluded.

**Results:**

The median age was 39 (range: 14–74) years and the majority were males (n = 128, 80.5%). Forty-nine patients (30.8%) presented with a recurrent fistula-in-ano. On flexible sigmoidoscopy, internal opening was seen in only 23 patients (14.4%). Furthermore, incidental findings of haemorrhoids (n = 5, 3.1%) and polyps (n = 7, 4.4%) were found. One patient (0.6%) had a healed anal fissure, 5 patients (3.1%) had inflamed mucosa and 2 patients (1.3%) had ulcers. Only two patients with inflamed mucosa were diagnosed to have Crohn’s disease on histology. Therefore, flexible sigmoidoscopy was not helpful in the majority to locate the internal opening. Only two patients had evidence of an underlying aetiology, which was Crohn’s disease. However, they had recurrent complex fistulae and other associated symptoms. Therefore, flexible sigmoidoscopy may be reserved for selected group of patients with symptoms of an underlying aetiology.

## Introduction

Although the majority of anal fistulae are cryptoglandular, in some, an underlying aetiology such as inflammatory bowel disease (i.e. Crohn’s disease and ulcerative colitis), diverticulitis, tuberculosis and cancer may be present [1]. Accurately determining the anatomy of the fistula and any underlying condition prior to treatment helps to achieve cure and minimises recurrences [2–4].

Prior to definitive treatment, flexible sigmoidoscopies are performed routinely in some units for patients with fistula-in-ano to detect an underlying aetiology. However, routine flexible sigmoidoscopy may result in higher cost, especially in a setting with limited resources and subjects patients to discomfort and sometimes complications due to endoscopy [5, 6].

Therefore, the aim of our study was to evaluate the yield of routine flexible sigmoidoscopy in patients presenting with fistula-in-ano.

## Main text

### Materials and methods

This is a retrospective analysis of 159 consecutive patients who presented to the University Surgical Unit of National Hospital of Sri Lanka, with a fistula-in-ano between January 2014 and September 2018. Ethical approval was obtained from Ethical Review Committee of National of Sri Lanka to conduct the study. All patients gave informed written consent to participate in this study. All patients were evaluated with a rectal examination, flexible sigmoidoscopy and examination under anaesthesia. Those with a diagnosed underlying aetiology such as Crohn’s disease, diverticulitis and colorectal cancer and presented with a fistula-in-ano as a complication were excluded from the study.

All the flexible sigmoidoscopy procedures were carried out by a consultant colorectal surgeon or a trainee under supervision. A phosphate enema was given 30 min before flexible.

Sigmoidoscopy in all patients. Patients were positioned in the left lateral position. The procedure was done with a standard 60-cm fibre optic flexible sigmoidoscope. At the end of the procedure, examination of the anal canl was performed using retroflexion and proctoscopy. Details related to the procedure such the extent of the bowel visualised, findings, quality of preparation and patient details were recorded in a computer based database. Analysed data were expressed in terms of frequencies and percentages.

### Results

A total of 159 patients were included in the analysis. The majority were males 128 (80.5%). Median age was 39 (range: 14–74) years. Forty-nine (30.8%) patients presented with recurrent fistulae. Twenty-five patients (15.72%) presented with additional symptoms other than perianal discharge such as rectal bleeding (n = 2, 1.25%), constipation (n = 4, 2.51%) and lump at the anus (n = 6, 3.77%). The majority were trans-sphincteric tracts (n = 84, 52.8%) followed by inter-sphincteric (n = 43, 27.0%), superficial (n = 16, 10.1%) and supra-sphincteric (n = 7, 4.4%) tracts (Table 1).

**Table 1:** Demographic characteristics and types of fistula

	N	%
Age in years (Mean ± SD)	40 ± SD 13	
Gender		
Male	128	80.5
Female	31	19.5
Recurrent fistula		
Yes	49	30.8
No	110	69.2
Type of frimary fract		
Superficial	16	10.1
Inter-sphincteric	43	27.0
Trans-sphincteric	84	52.8
Supra-sphincteric	7	4.4
Extra-sphincteric	1	0.6
Horse shoe	1	0.6
Multiple tracts	1	0.6
Not classified	6	3.8
Number of internal openings		
1	155	97.5
2	4	2.5
Level of internal opening		
Below the dentate line	68	42.8
At the dentate line	67	42.1
Above the dentate line	7	4.4
Rectum	6	3.8
Not classified	11	6.9
Horse shoeing		
Inter-sphincteric	4	2.5
Infra-levator (in ischiorectal fossa)	3	1.9
Absent	152	95.6
Abscess		
Inter-sphincteric	8	5.0
Infra-levator	6	3.8
Supra-levator	1	0.6
Absent	144	90.6
Sphincter defects		
Present	34	21.4
Absent	125	78.6

Visualization was done up to the splenic flexure in the majority (n = 129, 81.1%). In ten patients, (6.3%) colon was visualised beyond the splenic flexure, in 13 patients (8.1%) colon was visualised up to the descending colon and in 7 patients (4.4%) colon could not be visualised beyond the sigmoid colon. In 8 patients (5.03%) there was poor bowel preparation and in 1 patient, the procedure was abandoned due to excessive pain.

Sigmoidoscopy findings of anus, rectum, sigmoid colon and the descending colon were evaluated. Internal opening of the fistula was only seen in 23 patients (14.4%), 2 of which were seen in the rectum. Furthermore incidental findings of haemorrhoids (n = 5, 3.1%) and polyps (n = 7, 4.4%) were found. One patient (0.6%) had a healed anal fissure, 5 patients (3.1%) had inflamed mucosa and 2 patients (1.3%) had ulcers (Table 2). A total of 139 patients (87.4%) had normal studies on flexible sigmoidoscopy. Of the five patients with inflamed mucosa, 2 were found to have histological evidence of Crohn’s disease while others had unremarkable histological findings. Patients with polyps and ulcers were benign and there was no evidence to suggest an underlying aetiology for fistula-in-ano.

**Table 2:** Breakdown of flexible sigmoidoscopy findings

	Anus	Rectum	Sigmoid colon	Descending colon
Normal	130 (81.8%)	147 (92.5%)	152 (95.6%)	145 (91.2%)
Internal opening of fistula	21 (13.2%)	2 (1.3%)	–	–
Anal fissure	1 (0.6%)	–	–	–
Haemorrhoids	5 (3.1%)	–	–	–
Polyp	1 (0.6%)	3 (1.9%)	1 (0.6%)	2 (1.3%)
Inflamed mucosa	–	5 (3.1%)	1 (0.6%)	2 (1.3%)
Ulcer	–	1 (0.6%)	1 (0.6%)	–
Not visualized	–	–	2 (1.3%)	10 (6.3%)

### Discussion

Fistula-in-ano with an underlying aetiology is challenging to manage and associated with high risk of recurrence. Repeated surgeries may lead to incontinence and reduced quality of life [7, 8]. Flexible sigmoidoscopy is performed for a number of anorectal symptoms for confirmation of diagnosis and exclusion of other underlying causes. Similarly, routine flexible sigmoidoscopy is performed in some units for evaluation of an underlying aetiology in fistula-in-ano. However, there is scarcity of data assessing the effectiveness of flexible sigmoidoscopy in detection of an underlying aetiology in fistula-in-ano. Performing unnecessary routine procedures subject patients to discomfort, risk of complications of endoscopy and may escalate cost on patient care especially in a resource limited setting [6].

The primary focus of this study was to assess the ability to diagnose an underlying aetiology with routine flexible sigmoidoscopy. The majority (n = 139, 87.4%) had no detectable abnormalities on flexible sigmoidoscopy while others had incidental findings. There were 5 patients who were found to have inflamed mucosa suspected of Crohn’s disease and only 2 patients were later diagnosed to have Crohn’s disease through histology while in the others histology was unremarkable. The two patients diagnosed to have Crohn’s disease presented with recurrent complex fistulae and other associated symptoms such as loose stools and abdominal pain. There is increasing prevalence of inflammatory bowel disease in the region [9, 10]. Therefore, excluding such underlying cause is important for the management.

Histological assessment of those with polyps and ulcers revealed benign findings. One patient presented with an anal fissure along with a fistula. The anal fissure was initially treated prior to attempting a flexible sigmoidoscopy and further follow up was unremarkable with no recurrence of symptoms or evidence of Crohn’s disease. Therefore, based on our findings, flexible sigmoidoscopy may be reserved for a selected group of patients with recurrent complex fistulae and associated symptoms of an underlying aetiology.

Flexible sigmoidoscopy was not useful in detecting internal opening in the majority. Only 23 (14.4%) internal openings were seen on flexible sigmoidoscopy, 2 of which were found in the rectum. However, examination under anaesthesia was done in all patients revealed an internal opening confirming a fistula. Multiple internal openings were present in 4 patients (2.5%) which however were not visualised on flexible sigmoidoscopy.

### Conclusion

This study revealed that flexible sigmoidoscopy was not helpful in finding the underlying aetiology in the majority of patients presenting with fistula-in-ano. The majority of flexible sigmoidoscopies were normal and most of the findings were incidental. Only two patients had evidence of an underlying aetiology which was Crohn’s disease. However, they had recurrent complex fistulae and other associated symptoms. Therefore, flexible sigmoidoscopy may be reserved for a selected group of patients with recurrent complex fistulae and associated symptoms of an underlying aetiology.

### Limitations

This is a retrospective study with a small sample size. However, the findings were systematically recorded in a computer database allowing accurate and easy retrieval of data.

## Data Availability

The datasets generated and analysed during the current study are available from the corresponding author on reasonable request.
